# *Klebsiella pneumoniae* species complex: From wastewater to the environment

**DOI:** 10.1016/j.onehlt.2024.100880

**Published:** 2024-08-17

**Authors:** Ilse Verburg, Lucia Hernández Leal, Karola Waar, John W.A. Rossen, Heike Schmitt, Silvia García-Cobos

**Affiliations:** a Wetsus, European Centre of Excellence for Sustainable Water Technology, 8900, CC, Leeuwarden, the Netherlands; b Department of Medical Microbiology and Infection Prevention, University Medical Center Groningen, University of Groningen, 9713, GZ, Groningen, the Netherlands; c Certe Medische Microbiologie Friesland, 8900, JA, Leeuwarden, the Netherlands; d Institute for Infectious Disease Control, National Institute for Public Health and the Environment (RIVM), 3721, MA, Bilthoven, the Netherlands; e Laboratorio de Referencia e Investigación en Resistencia a Antibióticos e Infecciones Relacionadas con la Asistencia Sanitaria, Centro Nacional de Microbiología, Instituto de Salud Carlos III, Majadahonda, Madrid, Spain; f CIBER de Enfermedades Respiratorias (CIBERES), Instituto de Salud Carlos III, Madrid, Spain

**Keywords:** *Klebsiella pneumoniae*, Wastewater pathway, Whole genome sequencing, Hospital, WWTP, Wastewater, Antibiotic resistance genes, Virulence genes, Plasmid replicon genes

## Abstract

*Klebsiella pneumoniae* plays a significant role in nosocomial infections and spreading antibiotic resistance, and therefore forms a major threat to public health. In this study, we investigated the role of the wastewater pathway in the spread of pathogenic bacteria and more specifically, in the spread of antibiotic resistant *Klebsiella pneumoniae* subspecies. Whole-genome sequencing was performed of 185 *K. pneumoniae* isolates collected from hospital, nursing home, and community wastewater, the receiving wastewater treatment plant (WWTP), and clinical isolates from the investigated hospital. *K. pneumoniae* isolates from different sources were not genetically related, except for WWTP influent (46.5%) and effluent (62.5%), revealing survival of bacteria from wastewater treatment. The content of antibiotic resistance (ARGs), virulence, and plasmid replicon genes differed between *K. pneumoniae* subspecies and their origin. While chromosomal *bla* genes were specific for each *K. pneumoniae* subspecies, *bla* genes predicted in plasmid contigs were found in several *K. pneumoniae* subspecies, implying possible gene transfer between subspecies. Transferable ARGs were most abundant in patients and hospital isolates (70%), but the average number of plasmid replicon genes per isolate was similar across all sources, showing plasmid content being more relevant than plasmid quantity. Most patient (90%) and hospital wastewater (34%) isolates were *K. pneumoniae* subsp. *pneumoniae*, and the yersiniabactin cluster genes *ybt, fyuA,* and *irp12* were only found in this subspecies, as were the IncFII(pECLA), IncHI2A, and IncHI2 plasmid replicon genes, suggesting the clinical origin of these type of plasmids.

## Highlights

• *K. pneumoniae* isolates survived the WWTP and were released into the environment.
• *K. pneumoniae isolates clustered together* based on location and species.
• *K. pneumoniae* subspecies differed in their gene content regarding AMR, virulence and plasmid genes.
• Most ARGs were found in patient and hospital *K. pneumoniae* subsp. *pneumoniae* isolates.
• The number of transferable ARGs present in *K. pneumoniae* isolates is associated to plasmid types.

## Graphical abstract

**Figure:**
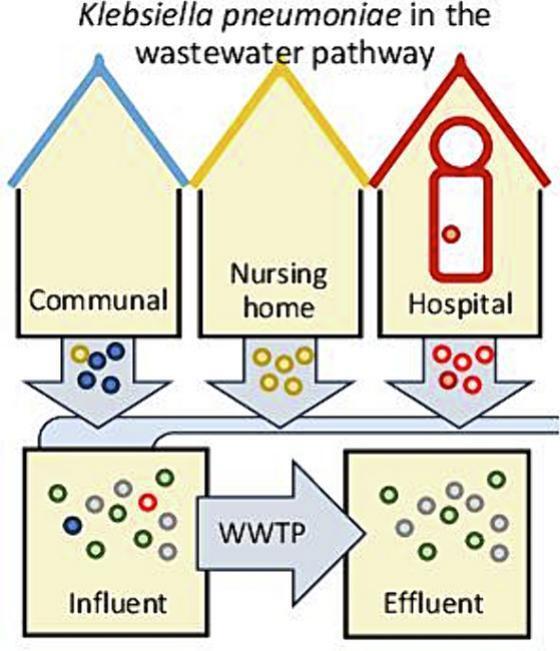

## 1 Introduction

The Gram-negative bacterium *Klebsiella pneumoniae* is an opportunistic pathogen linked to various infections, including urinary tract infections, respiratory infections, and bloodstream infections. It belongs to the ESKAPE pathogens, which play a crucial role in nosocomial infections, pathogenesis, and the spread of antibiotic resistance (AR) [1,2]. The *Klebsiella pneumoniae* complex includes seven species: *K. pneumoniae* subsp. *pneumoniae, K. quasipneumoniae* subsp. *quasipneumoniae, K. quasipneumoniae* subsp. *similipneumoniae, K. quasivariicola, K. africana, K. variicola* subsp. *tropica* and *K. variicola* subsp. *variicola* [3]. Several studies reported that *K. pneumoniae* subsp. *pneumoniae* is the most important clinical subspecies [[4], [5], [6]].

A One Health approach is crucial to combat AMR since the environment is connected with humans and animals and a possible reservoir of AMR. A better understanding of AMR reservoirs and AMR transmission between different One Health sectors can help to prevent it. *K. pneumoniae* is proposed to play a key role in spreading antibiotic resistance genes (ARGs) between the environmental and clinical settings [7]. *Klebsiella* spp. strains of clinical importance are possibly distinguishable by the presence of specific virulence genes and (transferable) ARGs. In addition, mobile genetic elements, such as plasmids, contribute to the pathogenicity of *K. pneumoniae,* as they are important vectors for the horizontal transfer of ARGs and virulence factors [8,9]. *K. pneumoniae* seems to develop or acquire antibiotic resistance mechanisms more easily than most bacteria, as most of the *K. pneumoniae* genome comprise accessory genes, which can encode specific virulence factors and antimicrobial-resistant enzymes and mechanisms [10].

*K. pneumoniae* species complex are widely distributed in the environment and are inhabitants of water, plants, animals and humans. However, recent studies have identified differentiation within *K. pneumoniae* genetic traits from different sources with minimal overlap noted [11].

Wastewater might play an essential role in disseminating faecal *K. pneumoniae* isolates to the environment, and research showed that wastewater treatment plants (WWTPs) are not completely removing ARGs and bacteria [12]. Our previous studies indeed showed that several bacterial species are decreased in the WWTP but are still present in the effluent [13,14]. However, it is unknown how isolates simply pass through the wastewater pathway, or different subpopulations are established at different sites depending on the conditions at specific sources.

This research investigates the genetic relatedness of *K. pneumoniae* isolates collected from wastewater from a hospital, a nursing home and a community, and the influent and effluent from the receiving WWTP from a Dutch municipality. Extra isolates from hospitalized patients with *K. pneumoniae* infections were examined. Additionally, the study examines the presence of antibiotic resistance, virulence, and plasmid replicon genes.

## 2 Material and methods

### 2.1 Origin of *K. pneumoniae* strains

*K. pneumoniae* strains were obtained from wastewater samples collected in our previous study [13]. Samples were collected monthly in 2017 from five different locations in Sneek, The Netherlands, including: community wastewater from an urban neighbourhood (80 households), wastewater from the main hospital in Sneek (300 beds), wastewater from the main nursing home in Sneek (220 beds), and water samples from the influent end effluent of a WWTP in Sneek. Two-liter water samples were collected flow-proportionally (WWTP) or time-proportionally (community, hospital and nursing home) for twenty-four hours using autosamplers. Grab samples were taken during a technical failure. One additional *K. pneumoniae* strain was obtained from the receiving surface water. Source and date of isolation are listed in supplementary table ST-1.

### 2.2 Culturing and selection of strains

*Klebsiella* spp. isolates were cultured as described in our previous work [13]: Water samples were filtered and incubated on Simmons citrate agar (Oxoid). Selected colonies were confirmed via MALDI-TOF MS and stored at −80 °C for further analysis. Three confirmed *K. pneumoniae* strains per location per sampling month were randomly selected for whole-genome sequencing (WGS). Only one strain was selected from grab samples. All *K. pneumoniae* isolates from the community wastewater (*n* = 22) were included. From one month, February, all *K. pneumoniae* isolates obtained from both influent and effluent of the WWTP (*n* = 10) were sequenced to increase the chance of finding related strains before and after the treatment. In addition, the medical laboratory Izore (Leeuwarden) provided twelve *K. pneumoniae* strains from hospitalized patients in 2017 from the investigated hospital. In total, 192 isolates were included for WGS (supplementary table ST-1).

### 2.3 DNA extraction and whole genome sequencing

Glycerol stocks were streaked twice onto blood agar plates (Mediaproducts) to obtain pure colonies. DNA was extracted using the DNeasy UltraClean Microbial Kit (Qiagen) and assessed using Qubit dsDNA BR Kit (ThermoFisher), targeting a minimum of 60 ng /μL [15]. DNA quality was measured using a Nanodrop™ 2000 Spectrophotometer (ThermoFisher). The library was prepared using Nextera XT DNA Library Prep (Illumina) [15] and sequencing was performed in a NextSeq (Illumina), using Nextseq mid-output cartridges (300 cycles). The quality of the assemblies were assessed using Quast [16].

### 2.4 Data analysis and visualization

Trimming and de novo assembly was performed using Qiagen CLC Bio Genomics Workbench 10.1.1. (Qiagen, Germantown, MD, USA).

The genetic relatedness of *Klebsiella* spp. isolates were studied using a gene-by-gene approach. Assembled genomes were imported into SeqSphere+ software v5.1.0 (Ridom GmnH, Münster, Germany), and a core-genome Multilocus Sequence Typing (cgMLST) scheme based on 2358 genes was applied. Phylogenetic relatedness was visualized using a minimum spanning tree, identifying genetically related isolates as Complex Types (CT) with a threshold of ≤15 allele differences.

ABRicate v0.8 (https://github.com/tseemann/abricate) was used to search for ARGs using the ResFinder database (last update 5 March 2019, [17]), plasmid replicon genes using the PlasmidFinder database (last update 5 March 2019, [18]), and virulence genes using an *in-house* database of virulence genes associated to *Klebsiella* spp. (last update 8 June 2018) [19]. A coverage of ≥70% and an identity of ≥90% was used to determine the presence of a gene. For *Klebsiella pneumoniae* subspecies, the MLST sequence type (ST), K-locus and O-locus were determined using Kleborate [20]. Contigs were analysed using a machine learning tool to classify them as chromosomes or plasmids (prediction). Mlplasmid [21] was used to predict the chromosomal or plasmid origin of contigs, thus determining the location of genes of interest for all isolates. This software employs a posterior probability threshold of 0.7 to produce confident results. In unexpected outcomes, the contig prediction was re-analysed using RFPlasmid [19].

Ordination plots were generated using Rstudio (version 2021.09.1) [22] with the method set to Principal Coordinates Analysis (PCoA) and the distance measure set to Jaccard to show the distance and clustering of the isolates based on the studied genes (ARGs, virulence genes and plasmid replicon genes) [23]. The pheatmap (RRID:SCR_016418) package was used to show both the cgMLST clustering and the clustering by the clinically important genes and gene content per subspecies.

## 3 Results and discussion

Of the 192 sequenced isolates, 185 belonged to the *K. pneumoniae* complex and were further studied. Summary data of sequenced isolates, source and date of isolation, subspecies, ST, O-locus and K-locus are shown in supplementary table ST-1. *K. pneumoniae* subsp. *similipneumoniae* (*n* = 60) was most abundant, followed by *K. variicola* subsp. *variicola* (*n* = 52), *K. pneumoniae* subsp. *pneumoniae* (*n* = 35), *K. pneumoniae* subsp. *quasipneumoniae* (*n* = 32), and *K. pneumoniae* subsp. *quasivariicola* (n = 6). The isolates belonged to 55 different STs, 11 different O-loci and 48 different K-loci. Forty-one isolates were not assigned to an ST. The most common STs were ST4756 (*n* = 18), ST138 (*n* = 15), ST3318 (*n* = 11), ST1255 (*n* = 10), and ST1485 (n = 10).

### 3.1 Separate clusters are formed by species and coincide with sources

**Fig. 1:**
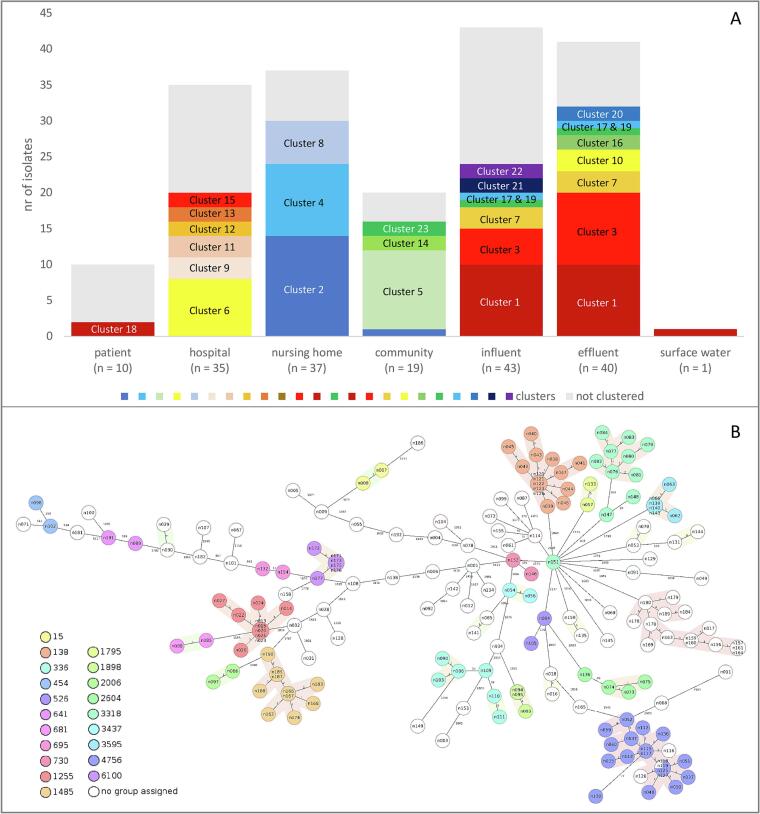
CgMLST clustering. A: bar plots showing the total number of isolates per location and the number of clustered isolates (≤ 13 allele differences). B: MST of all isolates coloured by ST, shading shows which isolates cluster together (≤ 15 allele differences). Patient and wastewater locations: only one isolate from community wastewater clustered with another source (nursing home, dark blue). Further, all clusters only contained isolates of the same source. WWTP and surface water locations: clusters 1, 3, 7, 17 and 19 were formed by isolates from both influent and effluent locations. Furthermore, one isolate from the surface water clustered with influent/effluent isolates (cluster 1). (For interpretation of the references to colour in this figure legend, the reader is referred to the web version of this article.)

We observed 23 groups of 2–20 isolates, 123 isolates (66%) were included in one of these groups based on cgMLST analysis (Fig. 1 and Supplementary Figs. SF-1 a-f). Genetically related isolates (0–13 allele differences) were occasionally obtained from single samples, but all groups with more than three isolates had isolates from more than one water sample. Clusters containing isolates from different water sources contained solely WWTP influent and effluent as sources, and in one case, also an isolate from surface water (Supplementary Table ST-2). Patient and wastewater isolates did not cluster (Fig. 1A, and Supplementary Fig. ST-1 a), except for one community wastewater isolate clustering with isolates from the nursing home wastewater (5–13 allele differences).

Clusters never contained isolates belonging to more than one ST and only cluster 1 (mainly ST4756) included three isolates that could not be assigned to a ST. Most clusters exclusively included isolates from the same location and the same species. Thus, the different wastewater sites seem to select for specific clones. ST526 (*K. quasipneumoniae* subsp. *quasipneumoniae*) was found once in hospital wastewater and once in effluent (149 allele differences), ST641 (*K. variicola* subsp. *variicola*) was found in hospital wastewater and in nursing home wastewater (326 allele differences), and ST3318 (*K. quasipneumoniae* subsp. *similipneumoniae*) was found in hospital wastewater (*n* = 8) and influent (*n* = 3) with 103–131 allele differences. In a similar study, *K. pneumoniae* isolates with the same ST were found in hospital wastewater and the receiving WWTP [24], but these isolates were not further analysed to establish the level of genetic relatedness. Analysis of 3482 *Klebsiella* genome sequences showed no relation between *K. pneumoniae* isolates from human, animal and environmental sources (rivers, ditches and ponds) [11]. However, this does not imply that *K. pneumoniae* from patients or the general population would not reach the environment. Research on ESBL-producing and carbapenem-resistant *K. pneumoniae* isolates revealed three *K. pneumoniae* river isolates that were indistinguishable from patient isolates collected in a similar time frame [25], indicating that pathogens from hospitalized patients can reach the environment. The high diversity between isolates belonging to *K. pneumoniae* results in a minor chance of finding related subspecies in different sources. We thus cannot exclude, that the lack of overlap between isolates from different sources in our study is due to a small sample size per location (<40 isolates per site). Alternatively, exclusively searching for resistant phenotypes will probably reduce diversity and increase the chances of finding related subspecies.

In this study, we found ST3318 (*K. quasipneumoniae* subsp. *similipneumoniae*) in both hospital wastewater and influent (Supplementary Table ST-1). However, these isolates did not cluster together by cgMLST analysis (Supplementary Fig. SF-2 a), which indicates the importance of performing analysis with high discriminatory power to investigate the genetic relatedness of bacteria.

### 3.2 *Klebsiella* spp. isolates survive wastewater treatment and reach the environment

We observed cgMLST clusters that were formed by both influent and effluent isolates (0–7 allele differences) (Fig. 1A, clusters 1, 3, 7, 17 and 19): these clusters included 62.5% (25/40) of the total effluent isolates and 46.5% (20/40) of the total influent isolates. In addition, the single isolate retrieved from surface water clustered with WWTP isolates (1 allele difference). This shows that *K. pneumoniae* clones survive the wastewater treatment and are released into the environment. None of the isolates from the WWTP clustered with isolates from the wastewater sources. Isolates might not survive the transport in the sewer pipes, or, more likely, the large diversity of isolates might have prevented identifying similar isolates in sources and WWTP influent. Previous studies showed that concentrations or relative abundances of several bacterial species decrease during wastewater treatment by 2 log CFU/L or by at least factor 10 (one log ratio), but all investigated species were still present in the effluent [13,14]. Here, in-depth sequence analysis confirms that bacteria survive the wastewater treatment since the species present in the effluent are genetically related to the species in the influent. Actually, the transport to the WWTP has more impact on the composition of the subspecies than the wastewater treatment.

### 3.3 CgMLST clusters are similar to hierarchical clusters based on antibiotic resistance, virulence and plasmid replicon genes

To investigate whether potentially mobile genes – i.e. ARGs, virulence and plasmid replicon genes - were shared between *K. pneumoniae* isolates from different locations, even if differing in STs, we performed clustering based on these genes solely (Supplementary Figs. S-2 a-e). This clustering was similar to the clustering identified by cgMLST analysis. Most isolates (92%) that clustered by cgMLST analysis, considering a threshold of ≤15 allele differences, also fell into one or two groups based on clustering by ARGs, virulence, or plasmid replicon genes, indicating that these genes share the same subspecies host distribution.

### 3.4 Genome location of genes and distribution over species

ARGs were predicted in chromosome and plasmid contigs (Table 1 and Table 2), those predicted in plasmid contigs were considered transferable. The *fos-* and *oqx-*genes were located on the chromosome and were commonly present in all *Klebsiella* subspecies, as previously described [26,27]. ARGs predicted in contigs of plasmid origin were more prevalent in *K. pneumoniae* subspecies *pneumoniae* isolates, which were related to patient and hospital wastewater origin (Supplementary Table ST-5). This subspecies is often identified as the most prevalent among *Klebsiella* spp. causing hospital and community-acquired infections [[4], [5], [6]], and thus a higher antibiotic exposure can increase the chance of acquiring ARGs.

**Table 1:** antibiotic resistance genes (without β-lactamase genes) and their predicted location using mlplasmids [21].

Antibiotic family	ARGs	No. genes total	No. genes predicted in plasmid contigs	No. genes predicted in chromosome contigs
Aminoglycosides	*aac3* *aac6* *aadA2* *ant3* *aph3* *aph6*	4 8 3 2 12 11	4 8 3 2 12 11	
Fosfomycin	*fosA* *fosA5* *fosA6*	127 16 30		127 16 30
Fluoroquinolones	*qnrB* *qnrS1*	10 3	10 2	1^a^
Macrolides	*ereA* *mphA*	6 5	6 5	
MDR efflux pump	*oqxA* *oqxB*	177 177		177 177
Phenicols	*catA2* *catB3*	6 3	6 3	
Sulfonamides	*sul1* *sul2*	15 12	15 12	
Tetracyclines	*tetA* *tetC* *tetD*	8 1 2	8 1 2	
Trimethoprim	*drfA*	19	19	

a The contig of this gene was predicted to be located on the chromosome both by mlplasmids (probability 0.999) and RFPlasmid (probability 0.997).

**Table 2:** β-lactamase genes and their predicted location using mlplasmids [21]. Unexpected locations are shown in red.

*bla* genes	No. genes total	No. genes predicted in plasmid contigs	No. genes predicted in chromosome contigs
*bla*_CTX-M-14_	1	1	
*bla*_CTX-M-15_	8	5	3
*bla*_DHA-1_	1	1	1
*bla*_LEN_	58	58	58
*bla*_OPK_	92	92	92
*bla*_OXA_	9	9	9
*bla*_SHV-1_	1		1
*bla*_SHV-106_	3		3
*bla*_SHV-110_	2		2
*bla*_SHV-12_	5	3	2
*bla*_SHV-145_	1		1
*bla*_SHV-172_	1		1
*bla*_SHV-178_	1		1
*bla*_SHV-187_	15	1	14
*bla*_SHV-27_	4		4
*bla*_SHV-36_	1		1
*bla*_TEM_	14	14	

Most beta-lactamase (*bla*) genes were on chromosomal contigs (Table 2). Some *bla*-genes were related to specific *Klebsiella* species (Supplementary Table ST-6). In fact, the detection of chromosomal *bla* genes has been proposed to identify *Klebsiella* species: *bla*_*SHV*_ for *K. pneumoniae*, *bla*_*OKP*_ for *K. quasipneumoniae* and *bla*_*LEN*_ for *K. variicola* [28].

Although *bla*_SHV_ is originally a core chromosomal gene in *K. pneumoniae*, four *bla*_SHV_ were in contigs predicted as plasmid ones by both mlplasmids and RFPlasmid in this study (Supplementary Table ST-7). Considering both posterior probabilities (0.865–0.959) obtained from mlplasmids and number of votes (0.593–0.686) from RFPlasmid, we can conclude that the predicted locations of these *bla*_SHV_ genes are correct. In addition, previous studies reported that *bla*_*SHV*_ has been mobilized from the chromosome at least twice through the IS26 transposase. This mobilization has integrated *bla*_SHV_ into various plasmid backbones, which have subsequently spread across different bacterial species [29].

*Bla*_CTX-M-15_*, bla*_OXA-1_ and *bla*_TEM-1B_ were found in multiple *K. pneumoniae* subspecies (Supplementary Table ST-6) and predicted mostly in plasmid contigs (Table 2). Nevertheless, three *bla*_CTX-M-15_ were in contigs predicted as chromosome contigs (Supplementary Table ST-7). Here, also both posterior probabilities (all 0.999) from mlplasmids, and the number of votes (0.911–0.997) from RFPlasmid indicate that these predictions are correct. In fact, the chromosomal integration of *bla*_CTX-M-15_ in *K. pneumoniae* was first reported in 2010 [30].

Virulence genes are important factors related to microbial pathogenicity. All identified virulence genes were predicted in chromosomal contigs and the majority were present in all *K. pneumoniae* isolates (Supplementary Table ST-3). Core virulence genes such as fimbriae types 1 and 3 and the siderophore enterobactin [31,32], involved in establishing opportunistic infections and contributing to biofilm formation, were partially found in >94% of the isolates. The yersiniabactin (*ybt*) gene cluster, an important alternative strategy for iron acquisition [33,34], was found in eleven *K. pneumoniae* subsp. *pneumoniae* isolates (Supplementary Table ST-1): six hospital isolates (ST336), three patient isolates (ST45, ST48 and ST231), one nursing home isolate (ST626), and one WWTP influent isolate (ST22). *Ybt* genes have been described in *Klebsiella* spp. isolates causing infections [3] and commonly related to ST11 isolates [35]. Kleborate output provides a virulence score based on the presence of yersiniabactin, colibactin and/or aerobactin reflecting the accumulation of loci contributing to hypervirulence [20,36]. The virulence score was 0 for all the isolates in this collection, except those eleven harbouring *ybt* genes, which virulence score was 1. Although the virulent potential may differ between *K. pneumoniae* isolates of different origin, the ubiquity of this bacteria and the presence of a core virulence genome are excellent characteristics to infect susceptible hosts and cause opportunistic infections [37].

Most isolates (85%) harboured the IncFIB(K) plasmid replicon gene (Supplementary Table ST-4), common in *Klebsiella* [38,39]. While many plasmid types were widespread, IncFII(pECLA), IncHI2A, and IncHI2 were exclusively found in *K. pneumoniae* isolates harbouring yersiniabactin genes. IncHI2 plasmids are linked to antibiotic resistance in *Salmonella* [40] and *K. pneumoniae* [41,42].

**Fig. 2:**
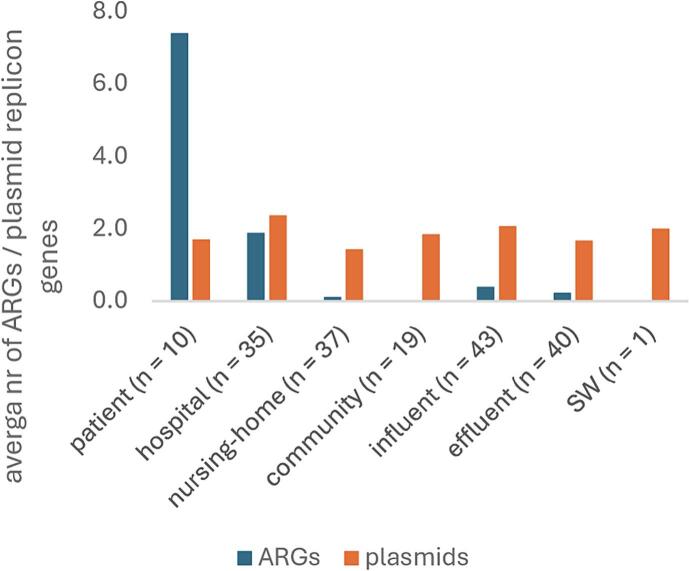
Average number of ARGs and plasmid replicon genes per location.

Plasmids play an important role in horizontal gene transfer (HGT) [[7], [8], [9]]. We calculated the average number of plasmid replicon genes per isolate and location, and observed similar gene counts (mean 1.4–2.4 plasmid replicon genes/isolate, Fig. 2). However, transferable ARGs were mainly found in patients (mean 7.4 ARGs/isolate) and hospital wastewater isolates (mean 1.9 ARGs/isolate). In a similar study, plasmid-borne ARGs were also more abundant in hospital wastewater than WWTP influent [43].

### 3.5 *K. pneumoniae* subspecies *pneumoniae* has the largest content of transferable ARGs

**Fig. 3:**
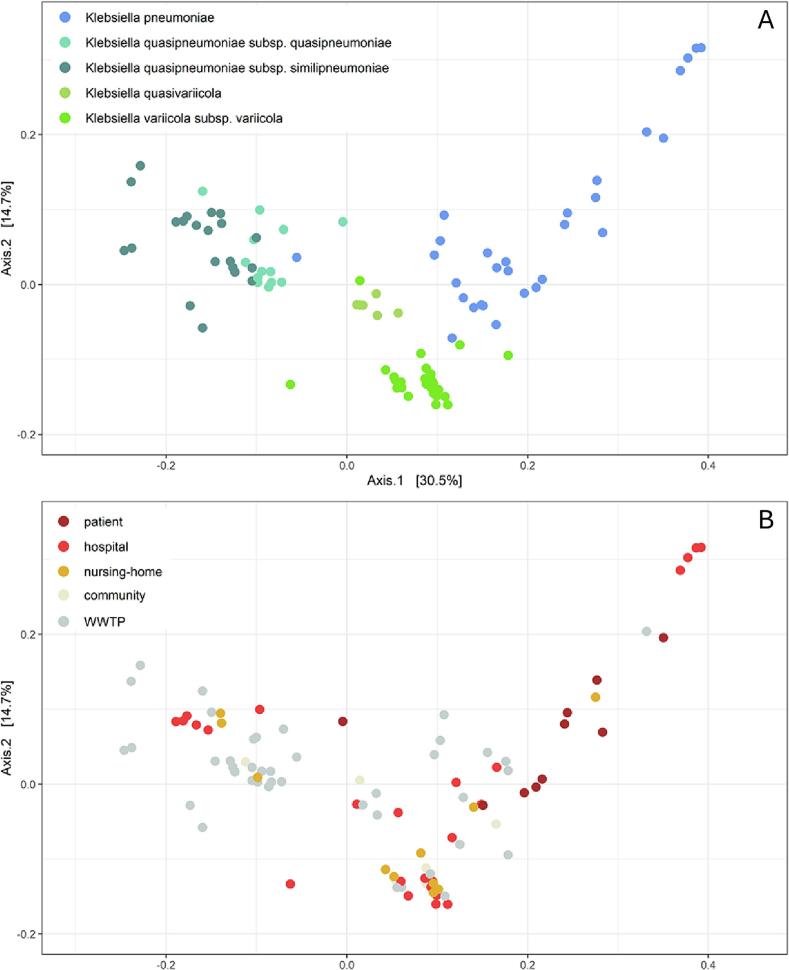
Ordination plots of isolates by their content of resistance genes, virulence genes and plasmid replicons, coloured by subspecies (A) and sources (B). Influent and effluent isolates are shown as WWTP. Plots were made as PCoA plots using the Jaccard method for the distance. Adonis test revealed that species explain 45% of the variation in distance, and 15% is explained by collection sources (*p* < 0.001).

PCoA analysis showed *K. pneumoniae* subspecies differ in their gene content concerning ARGs, virulence, and plasmid genes, as clustering by these genes was similarly to cgMLST analysis (Fig. 3A). Although patient isolates were not genetically related (Supplementary Table ST-1), PCoA revealed that most patient isolates were *K. pneumoniae* subsp. *pneumoniae* and grouped in one area (axis 1 > 0.15) of the PCoA plot (Fig. 3B). Ninety percent of the patient isolates, and 34.3% of the hospital isolates belonged to *K. pneumoniae* subsp. *pneumoniae*, accounting for 60% of these subspecies found in total (Supplementary Fig. SF-3). In a similar study, *K. pneumoniae* subsp. *pneumoniae* was also the most common *Klebsiella* subspecies in the hospital wastewater [43]. However, in this study *K. pneumoniae* subsp. *pneumoniae* was also the most prevalent in WWTP influent, which was not the case in our study.

Only one patient isolate, belonging to *K. quasipneumoniae* subsp. *quasipneumoniae,* was found in another area of the ordination plot.

No clustering according to wastewater sources was observed (Fig. 3B). Hospital wastewater could receive faecal material not only from hospitalized patients, but also from hospital staff and outpatients visiting the hospital. However, the contribution to hospital wastewater from outpatients and hospital staff is likely a small portion and the sampled community is only a minor part of the total, plus hospital staff and hospital visitors may be living in a different community. Thus, there is a minor chance to find isolates from these people in both the community wastewater and hospital wastewater.

**Fig. 4:**
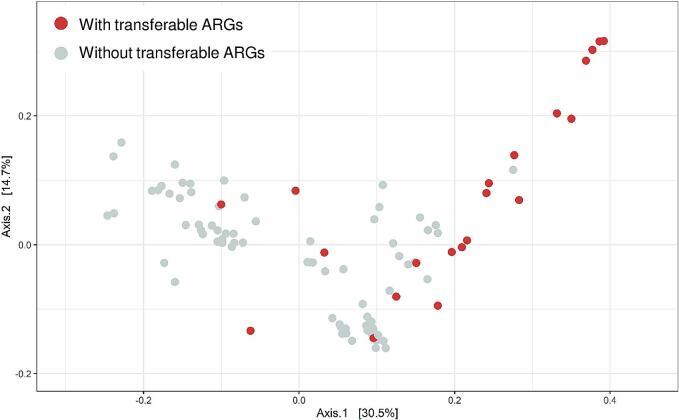
Ordination plot of isolates with and without transferable ARGs. Isolates harbouring one or more transferable ARGs are shown in red. Isolates that do not possess any transferable ARGs are shown in grey. Plots were made as PCoA plots using the Jaccard method for the distance. Adonis test revealed that 17% of the variation in distance is explained by transferable ARGs (p < 0.001). (For interpretation of the references to colour in this figure legend, the reader is referred to the web version of this article.)

In total, 23 isolates – mainly from patients (100%) and hospital wastewater (∼73%) – harboured transferable ARGs. In Fig. 4, these isolates clustered on one side of the PCoA plot (axis 1 > 0.15), in which also most of the patient isolates and all the *K. pneumonia* subsp. *pneumoniae* are displayed (Fig. 3A and B). These results indicate that transferable ARGs can distinguish isolates from clinical origin from other *K. pneumoniae* isolates. Moreover, it may indicate a major exposure to the “causative agent”: antibiotics.

## 4 Conclusions

The distinct subspecies of the *K. pneumoniae* species complex per site suggest that the different sites may be ‘selecting’ for different *Klebsiella* subcommunities, in which isolates of the same ST are conserved. While hospital wastewater and patient isolates were not detected in the downstream wastewater pathway, several isolates, including *K. pneumoniae* subsp. *pneumoniae* were found to survive the wastewater treatment process. Therefore, antibiotic resistant bacteria of concern to public health may end up into the environment via the wastewater pathway, evidencing the importance of including different One Health sectors to better understand the spread of AMR.

Clustering based on cgMLST analysis and hierarchical clustering by ARGs, virulence genes or plasmid replicon genes was similar, suggesting that these genes are differently distributed between *K. pneumoniae* subspecies. Most of the clinical isolates belonged to *K. pneumoniae* subsp. *pneumoniae*, and these subspecies carried the majority of potentially transferable ARGs. Nevertheless, potentially transferable *bla* genes were found in more than one subspecies, possibly resulting from gene transfer between different subspecies. ARGs located in contigs of predicted plasmid origin were mostly found in isolates related to patients or hospital wastewater. However, clinical isolates did not possess more plasmid replicon genes compared to non-clinical isolates, demonstrating that the type of plasmid, rather than the number of plasmids, is important. In addition to transferable ARGs, we identified virulence genes of clinical relevance, such as *ybt, fyuA,* and *irp1 + 2*, and the plasmid replicon genes IncFII(pECLA), IncHI2A and IncHI2, which might be of clinical importance. This study provides insights into the distribution and genetic content of *K. pneumoniae* subspecies isolates in the wastewater pathway.

## Funding

This work was performed in the cooperation framework of Wetsus, European Centre of Excellence for Sustainable Water Technology (www.wetsus.nl). Wetsus is co-funded by the Dutch Ministry of Economic Affairs and Ministry of Infrastructure and Environment, the European Union Regional Development Fund, the Province of Fryslân and the Northern Netherlands Provinces. This work was partly supported by the INTERREG VA (202085) funded project EurHealth−1 Health, part of a Dutch–German cross-border network supported by the 10.13039/501100000780European Commission, the 10.13039/501100002999Dutch Ministry of Health, Welfare and Sport (VWS), the 10.13039/501100007170Ministry of Economy, Innovation, Digitalisation, and Energy of the German Federal State of North Rhine-Westphalia and the German Federal State of Lower Saxony. S. G-C was recipient of a grant from the Research Talent Attraction Program - Modality 1 funded by Dirección General de Investigación e Innovación, Consejería de Educación e Investigación, Comunidad de Madrid (CAM) and by the Instituto de Salud Carlos III (ISCIII) (References 2018-T1/BMD-11174 and 2022-5 A/BMD-24243).

## CRediT authorship contribution statement

**Ilse Verburg:** Writing – original draft, Visualization, Methodology, Investigation, Formal analysis, Data curation. **Lucia Hernández Leal:** Writing – review & editing, Supervision, Project administration, Funding acquisition. **Karola Waar:** Supervision, Resources, Investigation. **John W.A. Rossen:** Writing – review & editing, Supervision, Resources, Funding acquisition. **Heike Schmitt:** Writing – review & editing, Supervision, Methodology, Formal analysis, Conceptualization. **Silvia García-Cobos:** Writing – review & editing, Visualization, Supervision, Software, Methodology, Investigation, Formal analysis, Conceptualization.

## Declaration of competing interest

The authors declare no conflict of interest. The funders had no role in the design of the study; in the collection, analyses, or interpretation of data; in the writing of the manuscript, or in the decision to publish the results.

## Data Availability

Short read whole-genome sequencing data are available in the European Nucleotide Archive (ENA) under project number PRJEB72890. The data that support the findings of this study are openly available in figshare at https://doi.org/10.6084/m9.figshare.25304302.v1 (Antibiotic Resistance Genes (ResFinder)), https://doi.org/10.6084/m9.figshare.25304527.v1 (Virulence genes (VFDB)), https://doi.org/10.6084/m9.figshare.25304545.v1 (Virulence genes (in-house database)), https://doi.org/10.6084/m9.figshare.25305715.v1 (Plasmid replicon genes (PlasmidFinder)), https://doi.org/10.6084/m9.figshare.25305730.v1 (Kleborate results), https://doi.org/10.6084/m9.figshare.25305748.v1 (MLplasmid results), https://doi.org/10.6084/m9.figshare.25305850.v1 (Assembly report (quast software)), and https://doi.org/10.6084/m9.figshare.25306006.v1 (RFPlasmid results).
