# Supplementary material for: Klebsiella pneumoniae species complex: From wastewater to the environment
Source: One Health. 2024 Aug 17;19:100880. doi: 10.1016/j.onehlt.2024.100880 (PMC11387367; doi:10.1016/j.onehlt.2024.100880)
Supplement: Supplementary tables and figures [file mmc1.docx]

# Supplementary material

| *Supplementary Table ST1: The sequenced strains, source, month of isolation, K. pneumoniae subspecies type, sequence type (ST), O-locus, K-locus, and cgMLST cluster. The 11 strains that contained yersiniabactin virulence genes are coloured in red.* |
| --- |
| ** |

|  |
| --- |
|  |

| *Supplementary figure SF1-a: minimum spanning tree based on cgMLST. Isolates are coloured by sources. Shading shows which isolates cluster together.* |
| --- |
| 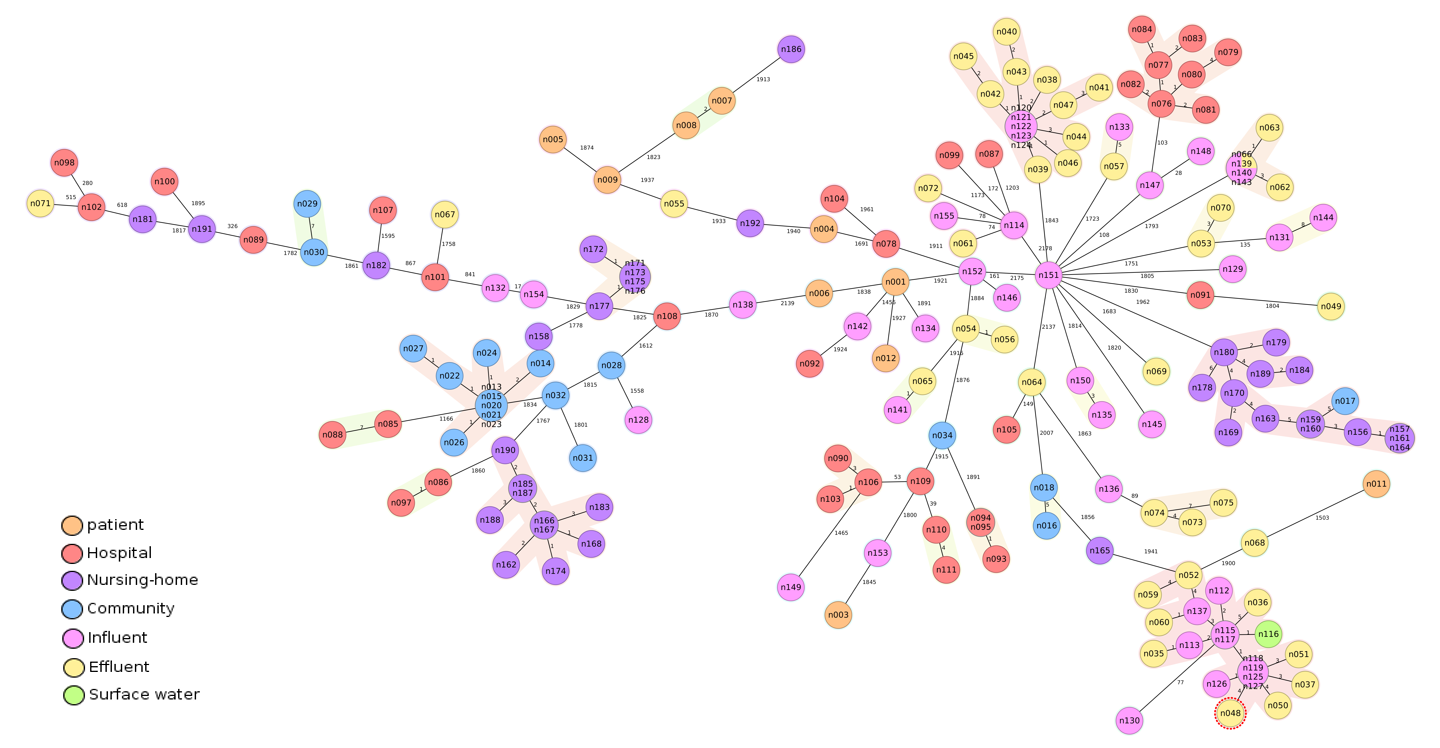 |

| *Supplementary figure SF1-b: minimum spanning tree based on cgMLST. Isolates are coloured by species. Shading shows which isolates cluster together.* |
| --- |
| 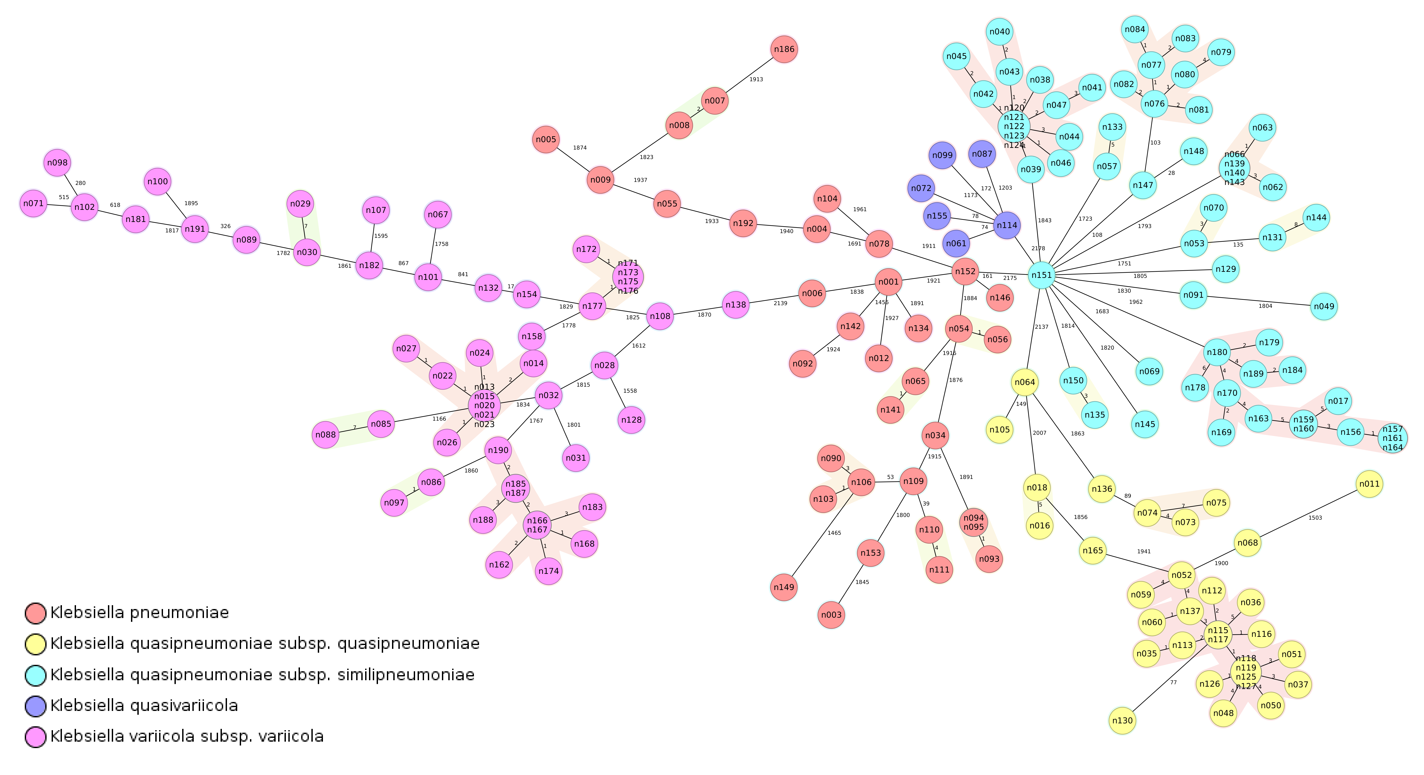 |

| *Supplementary figures SF1-c-f: minimum spanning tree based on cgMLST per Klebsiella species. Isolates are coloured by STs. Shading shows which isolates cluster together.* |
| --- |
| 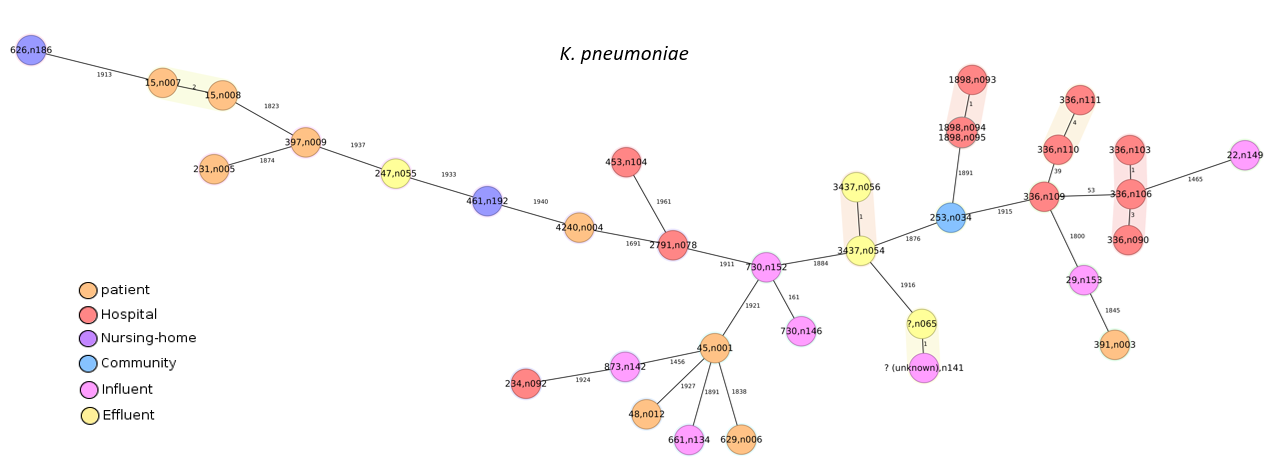 |
| 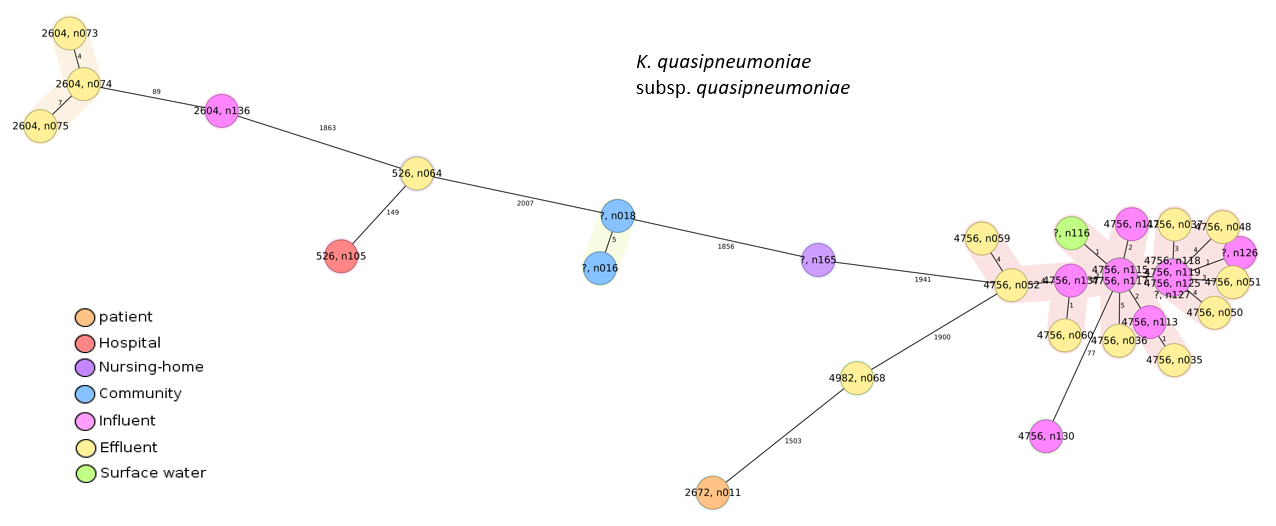 |
| 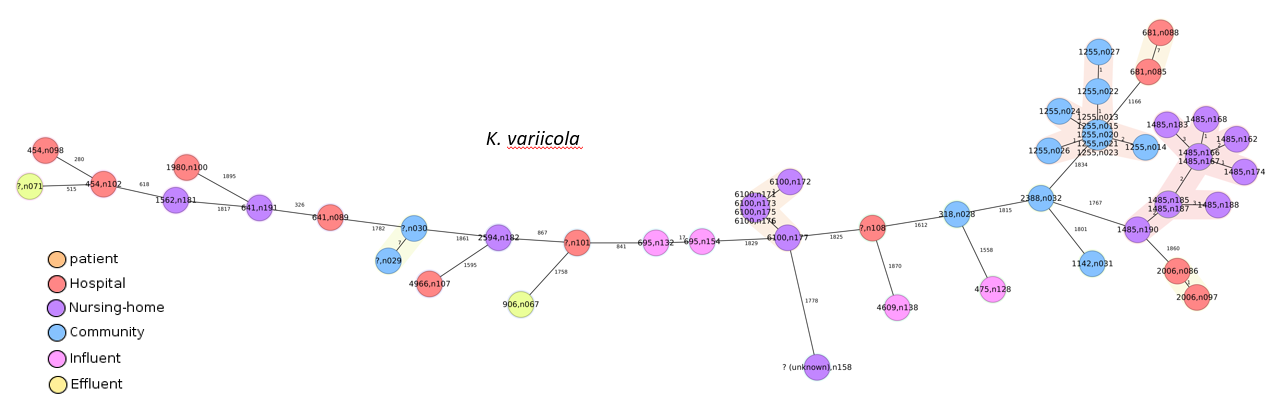 |
| 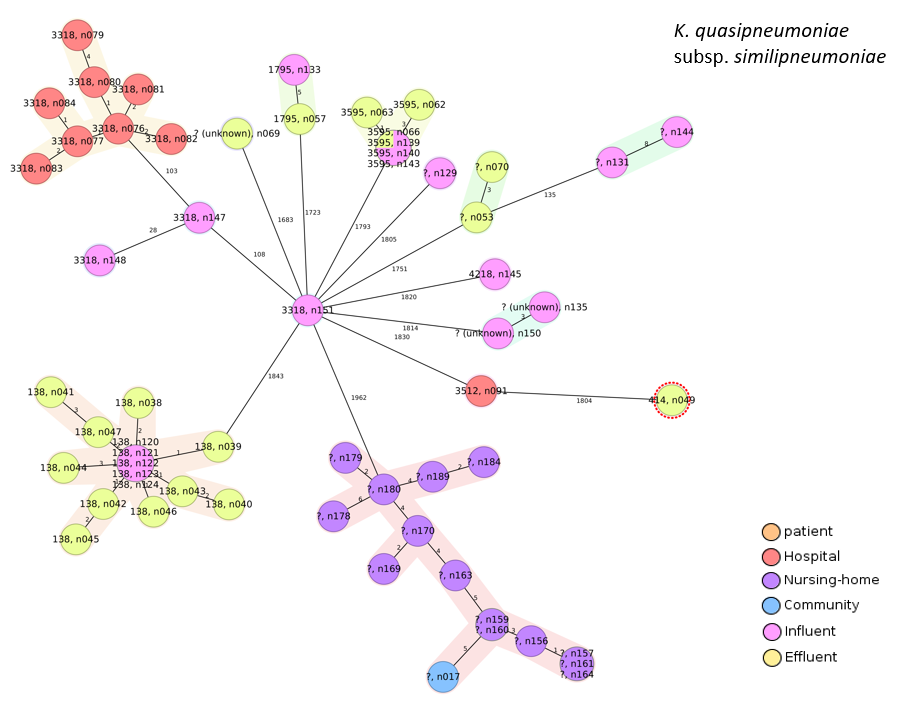 |

| *Supplementary Table ST2: The cgMLST clusters, nr of isolates, nr of samples from which isolates were obtained, allele difference range within the cluster, ST, subspecies and source(s). clusters consisting of strains from only one sampling day are indicated in red.* | | | | | | |
| --- | --- | --- | --- | --- | --- | --- |
| **cgMLST cluster** | **nr of isolates** | **Nr of samples** | **allele difference range** | **ST** | **subspecies** | **Source(s)** |
| **1** | 20 | 13 | 0-12 | ST4756 (n = 17) + NA (n = 3) | *K. quasipneumoniae subsp. quasipneumoniae* | Influent & effluent |
| **2** | 15 | 10 | 0-13 | NA | *K. quasipneumoniae subsp. similipneumoniae* | Nursing home & community |
| **3** | 15 | 3 | 0-7 | ST138 | *K. quasipneumoniae subsp. similipneumoniae* | Influent & effluent |
| **4** | 10 | 7 | 0-8 | ST1485 | *K. variicola subsp. variicola* | Nursing home |
| **5** | 10 | 6 | 0-3 | ST1255 | *K. variicola subsp. variicola* | Community |
| **6** | 8 | 6 | 1-7 | ST3318 | *K. quasipneumoniae subsp. similipneumoniae* | Hospital |
| **7** | 6 | 5 | 0-4 | ST3595 | *K. quasipneumoniae subsp. similipneumoniae* | Influent & effluent |
| **8** | 6 | 4 | 0-2 | ST6100 | *K. variicola subsp. variicola* | Nursing home |
| **9** | 3 | 3 | 1-4 | ST336 | *K. pneumoniae subsp. pneumoniae* | Hospital |
| **10** | 3 | 1 | 4-8 | ST2604 | *K. quasipneumoniae subsp. quasipneumoniae* | Effluent |
| **11** | 3 | 2 | 0-1 | ST1898 | *K. pneumoniae subsp. pneumoniae* | Hospital |
| **12** | 2 | 2 | 7 | ST681 | *K. variicola subsp. variicola* | Hospital |
| **13** | 2 | 2 | 1 | ST2006 | *K. variicola subsp. variicola* | Hospital |
| **14** | 2 | 2 | 7 | NA | *K. variicola subsp. variicola* | Community |
| **15** | 2 | 1 | 4 | ST336 | *K. pneumoniae subsp. pneumoniae* | Hospital |
| **16** | 2 | 2 | 1 | ST3437 | *K. pneumoniae subsp. pneumoniae* | Effluent |
| **17** | 2 | 2 | 1 | NA | *K. pneumoniae subsp. pneumoniae* | Influent & effluent |
| **18** | 2 | 1 | 3 | ST15 | *K. pneumoniae subsp. pneumoniae* | Patient |
| **19** | 2 | 2 | 5 | ST1795 | *K. quasipneumoniae subsp. similipneumoniae* | Influent & effluent |
| **20** | 2 | 2 | 3 | NA | *K. quasipneumoniae subsp. similipneumoniae* | Effluent |
| **21** | 2 | 2 | 8 | NA | *K. quasipneumoniae subsp. similipneumoniae* | Influent |
| **22** | 2 | 2 | 3 | NA | *K. quasipneumoniae subsp. similipneumoniae* | Influent |
| **23** | 2 | 1 | 5 | NA | *K. quasipneumoniae subsp. quasipneumoniae* | Community |

### Supplementary figure SF2: clustering and gene presence per subspecies

| 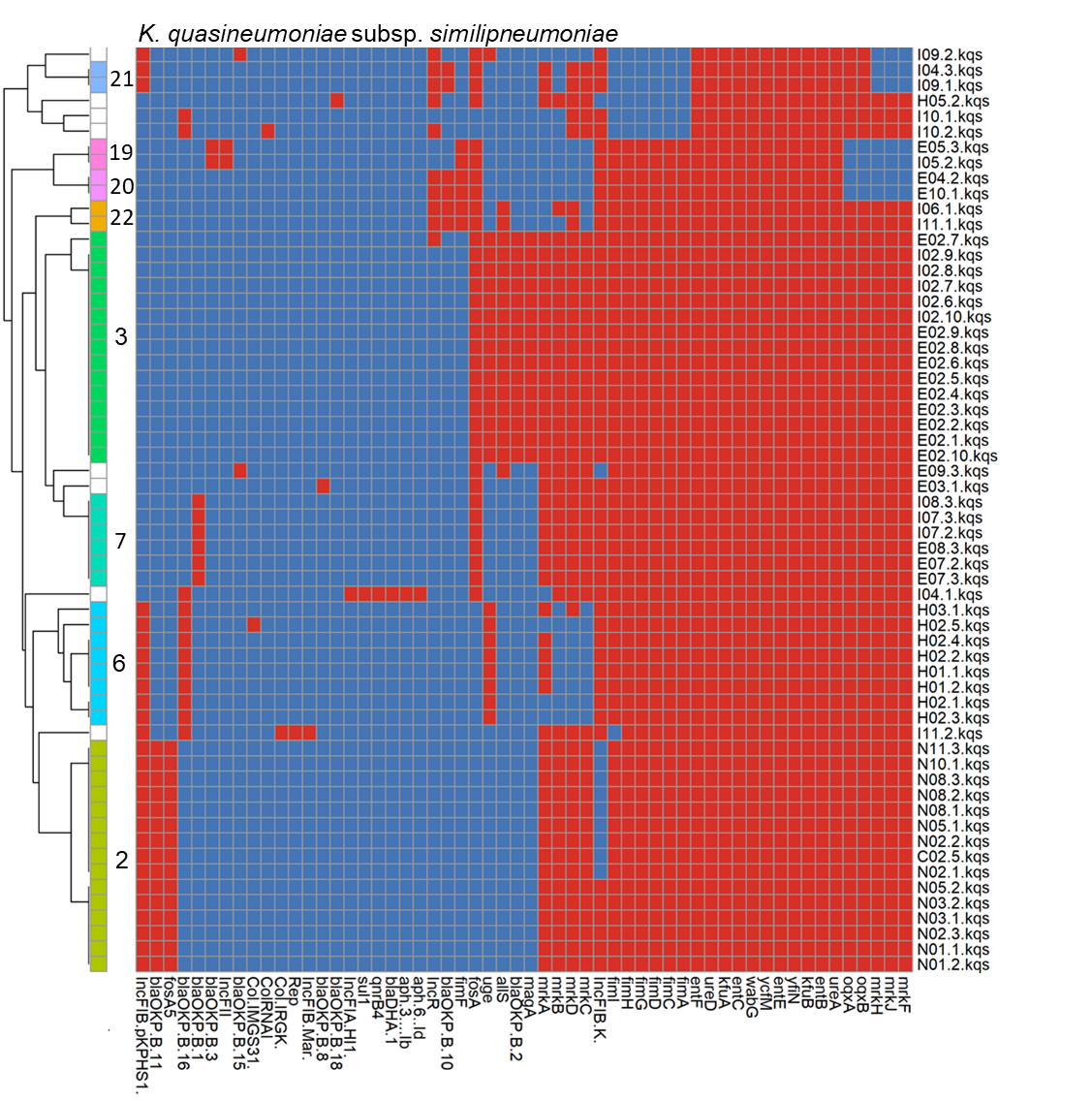 |
| --- |
| *Supplementary figure SFII-a: clustering and genes found in K. quasipneumoniae subsp. similipneumoniae. Red = gene is present, blue = gene is not present. The dendogram on the left side shows how isolates are clustered by the ARGs, VF genes and plasmid replicon genes (method used: binary). The bar shows the clustering by cgMLST; different colours are different clusters. no colour means the isolate did not cluster with another isolate.* |

| 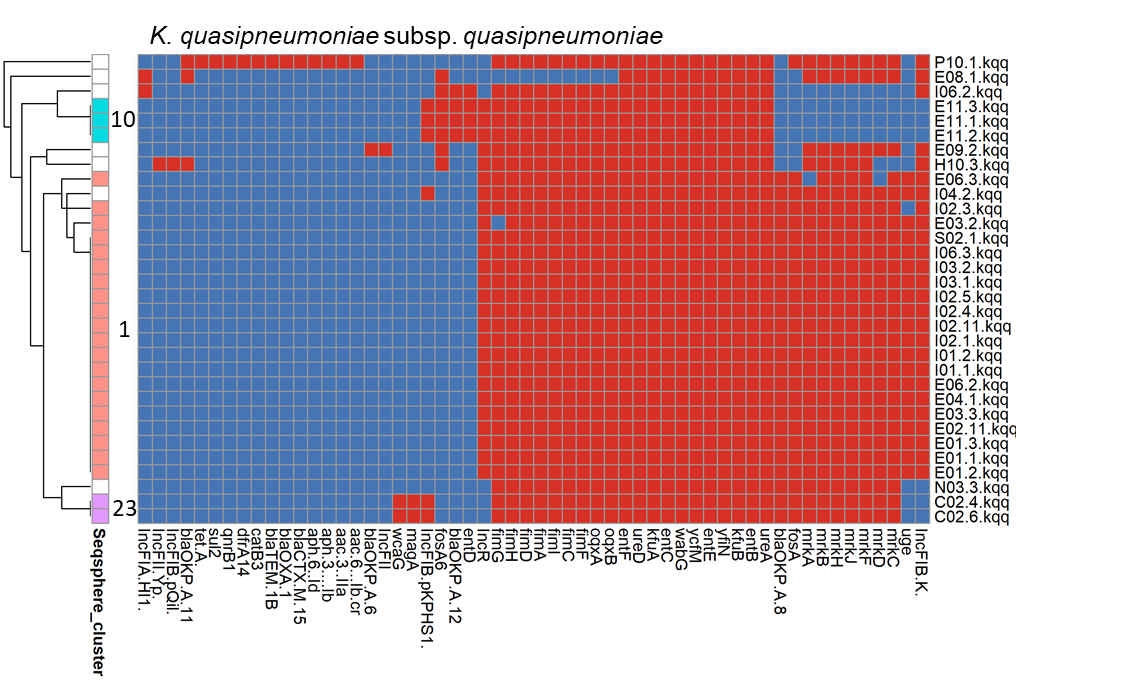 |
| --- |
| *Supplementary figure SF2-b: clustering and genes found in K. quasipneumoniae subsp. quasipneumoniae. Red = gene is present, blue = gene is not present. The dendogram on the left side shows how isolates are clustered by the ARGs, VF genes and plasmid replicon genes (method used: binary). The bar shows the clustering by cgMLST; different colours are different clusters. no colour means the isolate did not cluster with another isolate.* |

| 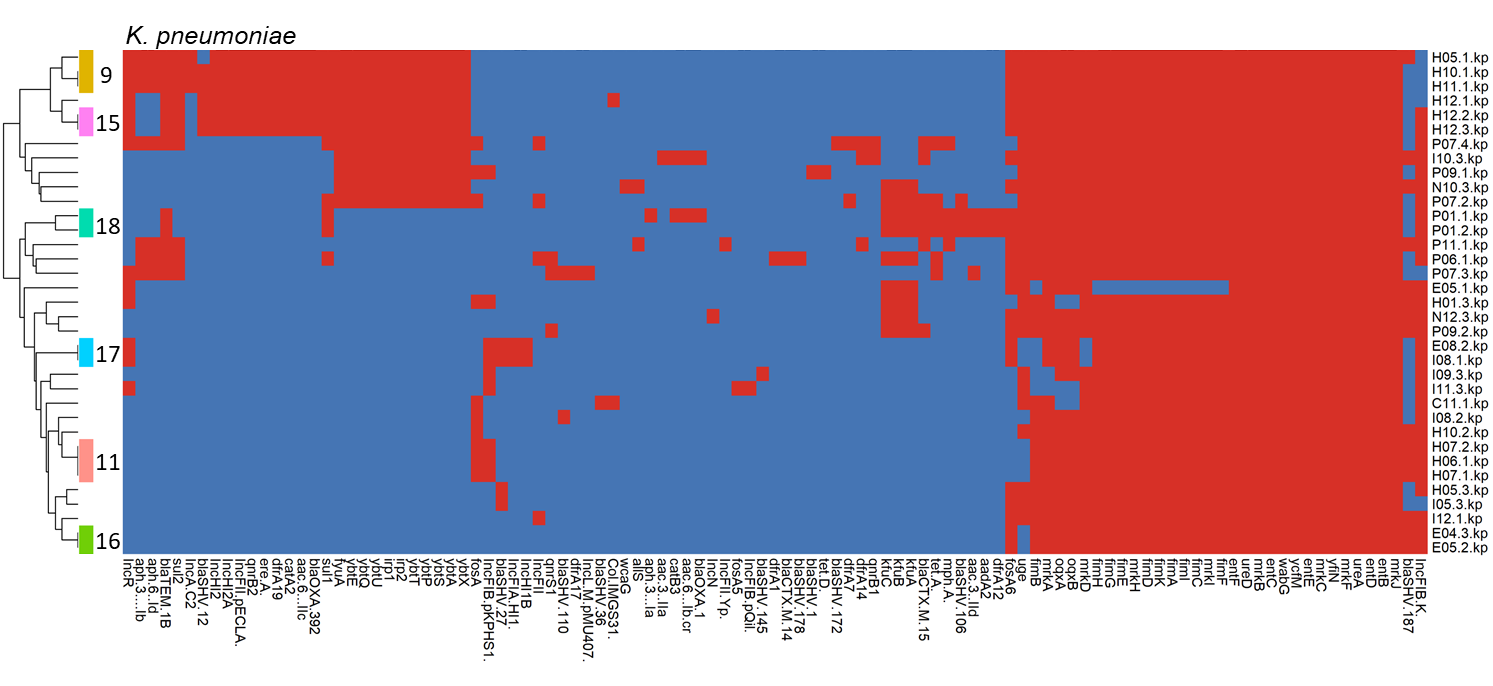 |
| --- |
| *Supplementary figure SF2-c: clustering and genes found in K. pneumoniae* subsp. *pneumoniae. Red = gene is present, blue = gene is not present. The dendogram on the left side shows how isolates are clustered by the ARGs, VF genes and plasmid replicon genes (method used: binary). The bar shows the clustering by cgMLST; different colours are different clusters. no colour means the isolate did not cluster with another isolate; none of the K. quasivariicola subspecies were clustered.* |

| 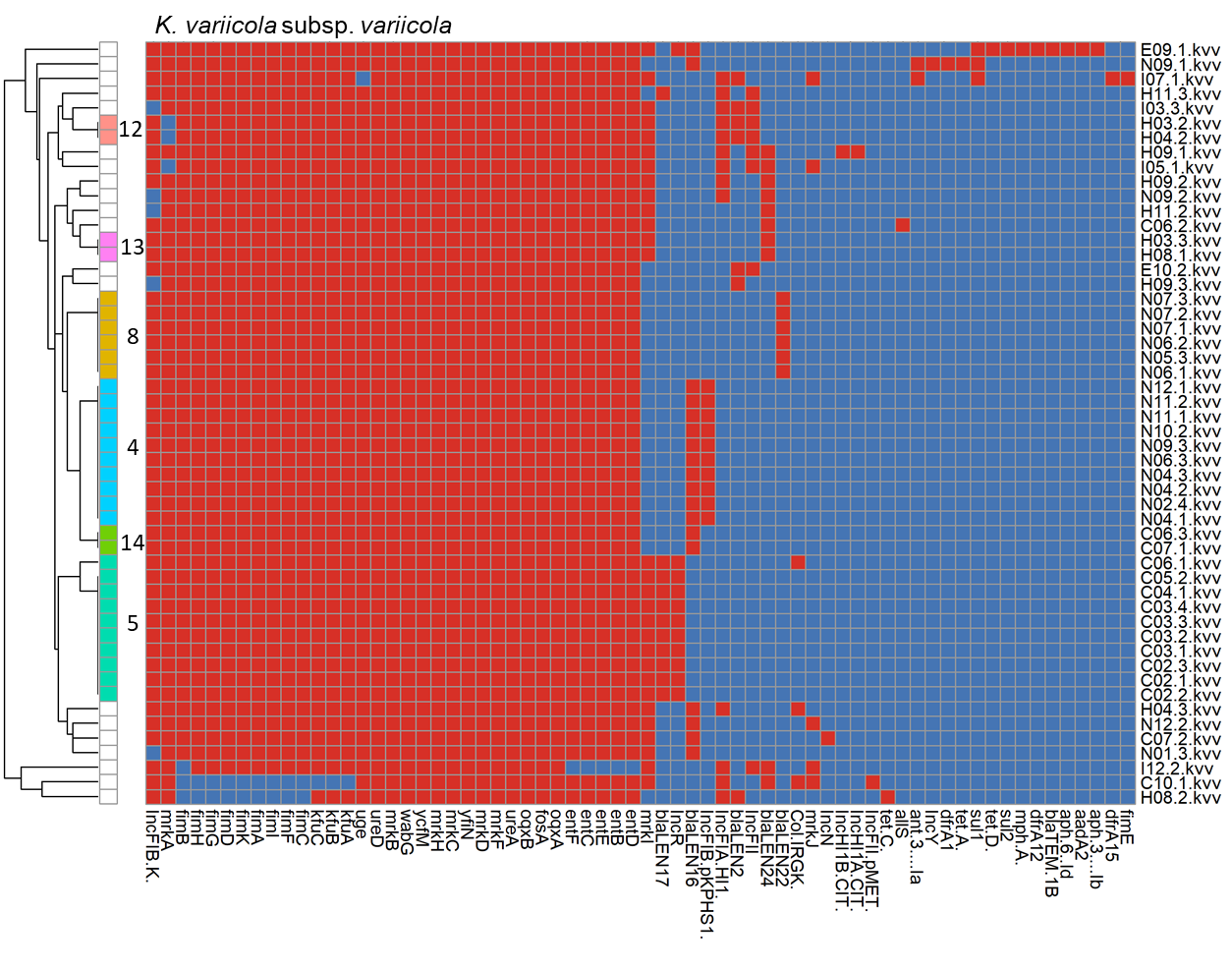 |
| --- |
| *Supplementary figure SF2-d: clustering and genes found in K. variicola* subsp. *variicola. Red = gene is present, blue = gene is not present. The dendogram on the left side shows how isolates are clustered by the ARGs, VF genes and plasmid replicon genes (method used: binary). The bar shows the clustering by cgMLST; different colours are different clusters. no colour means the isolate did not cluster with another isolate.* |

| 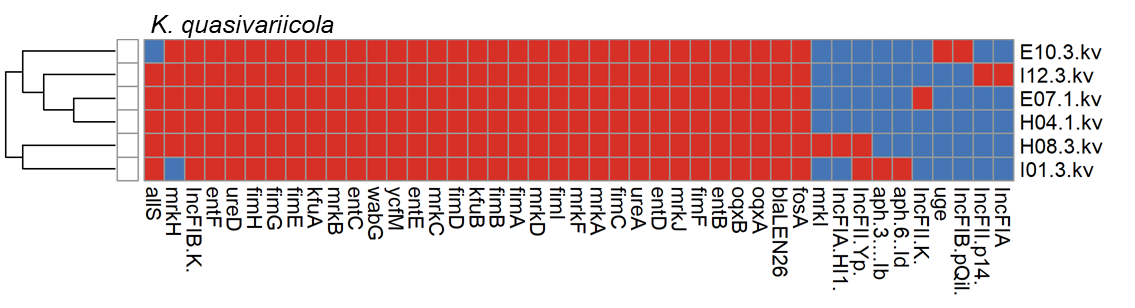 |
| --- |
| *Supplementary figure SF2-e: clustering and genes found in K. quasivariicola. Red = gene is present, blue = gene is not present. The dendogram on the left side shows how isolates are clustered by the ARGs, VF genes and plasmid replicon genes (method used: binary). The bar shows the clustering by cgMLST; different colours are different clusters. no colour means the isolate did not cluster with another isolate.* |

### Supplementary figure SF3: distribution of *K. pneumoniae* subspecies over the locations.

| 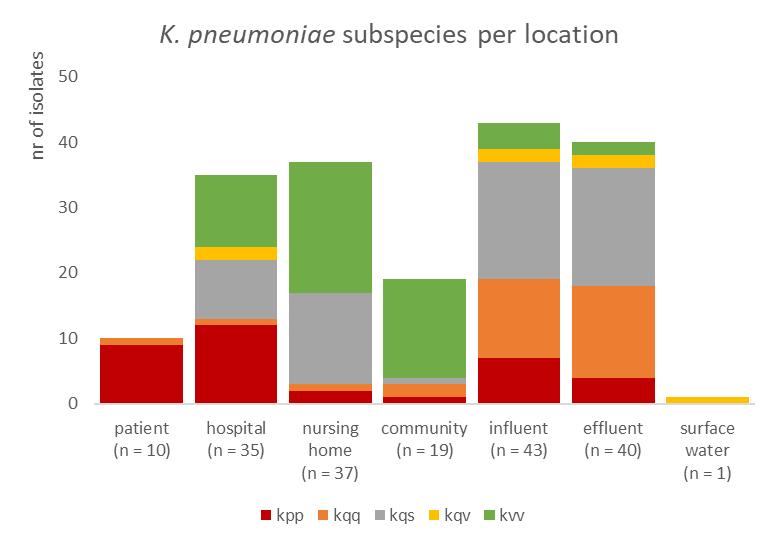 |
| --- |
| *Supplementary figure SF3: distribution of K. pneumoniae subspecies over the locations. kp = K. pneumonia subsp. pneumoniae; kqq = K. quasipneumoniae subsp. quasipneumoniae; kqs = K. quasipneumoniae subsp. similipneumoniae; kq = K. quasivariicola; kvv = K. variicola subsp. variicola* |

Supplementary Table ST3: distribution VF genes over Klebsiella subspecies.

Supplementary Table ST4: distribution plasmid replicon genes over Klebsiella subspecies.

*Two contigs containing IncFIB(K) were twice predicted as chromosomal by mlplasmids (posterior probability 0.511 and 0.772), but they were predicted as plasmid by RFPlasmid (votes 0.896 and 0.695). One contig containing IncR was predicted as chromosomal by mlplasmids (posterior probability 0.546), but it was as plasmid by RFPlasmid (votes 0.982). IncY was predicted to be located onto the chromosome by both mlplasmids (posterior probability = 0,809) and RFPlasmid (votes = 0,664).

Supplementary Table 5: distribution AMR genes over Klebsiella subspecies. Predicted chromosomal genes are shown in bolt.

Supplementary Table 6: distribution bla-genes over Klebsiella subspecies. Predicted chromosomal genes are shown in bolt.

*Three genes were predicted to be located on plasmid

**one gene was predicted to be located on plasmid

Supplementary Table 7: posterior probability (mlplasmids) and nr. of votes (RFPlasmid) of unexpected distributed bla-genes. Six isolates with unexpected distributed bla-genes contain yersiniabactin virulence genes (shown in red)

| Isolate | Source | Β-lactamase gene | Predicted location of contig | Posterior probability mlplasmids | Nr. Of votes RFPlasmid |
| --- | --- | --- | --- | --- | --- |
| P07.2 kp | Patient | *bla*_CTX-M-15_ | Chromosome | 0,999 | 0,996 |
| P09.2 kp | Patient | *bla*_CTX-M-15_ | Chromosome | 0,999 | 0,997 |
| I10.3 kp | Influent | *bla*_CTX-M-15_ | Chromosome | 0,999 | 0,911 |
| H11.1 kp | Hospital | *bla*_SHV-12_ | Plasmid | 0,952 | 0,730 |
| H12.1 kp | Hospital | *bla*_SHV-12_ | Plasmid | 0,959 | 0,686 |
| H12.3 kp | Hospital | *bla*_SHV-12_ | Plasmid | 0,865 | 0,737 |
| H05.1.kp | Hospital | *bla*_SHV-187_ | Plasmid | 0,886 | 0,593 |
